# Fast Green FCF Improves Depiction of Extracellular Matrix in Ex Vivo Fluorescence Confocal Microscopy

**DOI:** 10.3390/life14101240

**Published:** 2024-09-28

**Authors:** Maja Carina Nackenhorst, Adrian Hummel, Maximilian Christian Koeller, Bernd Gollackner, Heinz Regele

**Affiliations:** 1 Department of Pathology, Medical University of Vienna, 1090 Vienna, Austria; 2 Department of Vascular Surgery, Medical University of Vienna, 1090 Vienna, Austria

**Keywords:** digital pathology, ex vivo fluorescence confocal microscopy, extracellular matrix, frozen sections

## Abstract

Rapid microscopic analysis of tissue is an essential diagnostic tool in oncological surgery. The gold standard for intraoperative histological tissue evaluation is frozen sections. However, frozen sections are prone to a variety of artefacts and require skilled staff and specialized lab equipment. A potential method for rapid intraoperative tissue evaluation that does not require fixation, freezing, or sectioning of the tissue is ex vivo fluorescence confocal microscopy (FCM). The visualization of the structurally important extracellular matrix (ECM) in conventional ex vivo FCM lags behind the standards of conventional histology. The objective of this study was to find a stain that would improve the depiction of the ECM to resemble FFPE H&E sections as closely as possible. Eleven different tissue stains were tested on 122 tissue samples submitted to the Department of Pathology at the Medical University of Vienna. This study was conducted on the RS-G4 Upright (Caliber I.D. Rochester, NY, USA, distributed in Europe by MAVIG GmbH, Munich, Germany). Fast Green FCF (FGFCF) in combination with acridine orange as a nuclear stain improved the visibility of the structural details of the ECM. Morphological details in FCM were equivalent or even superior to frozen sections in most analyzed categories. The addition of FGFCF to the conventional staining protocol improves the assessment of the ECM and analysis of fibrosis. The rapid staining protocol is compatible with an application in intraoperative microscopy.

## 1. Introduction

Rapid microscopic tissue analysis is a pillar of pathology and has been used for decades in the form of frozen sections for the quick intraoperative diagnosis of malignancy and the assessment of resection margins in tumor surgery. Frozen sections do, however, have marked disadvantages, such as a low quality due to freezing artefacts, especially in tissues with little tissue cohesion or lipid-rich tissues, and the need for extensive lab equipment and specially trained personnel, as well as the loss of the tissue for further investigations [1].

Fluorescence confocal microscopy (FCM) has, in the past few years, emerged as a relevant tool for microscopic tissue analysis that may serve as an ancillary method in rapid tissue diagnostics or potentially even replace it [2,3,4,5]. FCM is a fast virtual microscopy technology that produces digital microscopic images from tissue samples without prior fixation, freezing or sectioning.

In a previous study, we investigated whether FCM is a suitable tool for rapid tissue analysis of specimens rich in adipose tissue, such as breast tissue [6].

We found that FCM can be useful in the analysis of these specimens and the depiction of cellular nuclei was excellent and comparable to hematoxylin and eosin (H&E)-stained slides. However, we also noticed that the extracellular matrix (ECM) was generally less well defined than in conventional histology and showed a different texture. 

The ECM is an integral part of all tissues and consists of various proteins with an often fibrillary structure and functions as a stabilizing framework [7]. The amount and the histologically visible texture of the ECM is tissue-specific but highly variable among different areas of the body. Moreover, pathologic conditions like inflammation can trigger excess deposition of the ECM, a process that is called fibrosis. This may occur in almost all types of tissues and frequently leads to the impairment of organ function. Specific structures of a normal ECM are the basement membranes (BMs) that delineate epithelial cell compartments in many tissue types like the skin and various glandular organs. Beyond its functional role in defining tissue architecture and guiding cell growth, it is also of outstanding diagnostic importance in oncology. Many treatment choices depend on correctly identifying the level of invasiveness of the tumor. The basement membrane and the rest of the ECM should therefore be depicted in as much detail as possible. It should match the current gold standard for ECM evaluation, namely H&E-stained histology sections, as closely as possible.

In this follow-up study, we investigated potential staining options for a better depiction of the ECM that can both (1) be implemented in FCM and (2) be performed in a sufficiently short amount of time to be suitable for the application of FCM in rapid tissue assessment.

## 2. Materials and Methods

### 2.1. Patients

This study was approved by the local Ethics Committee (protocol number: 1872/2019). One hundred twenty-two tissue samples from 18 patients were included. Samples were anonymized without the possibility of tracing them back to the respective patient.

### 2.2. Tissue Specimen

Seven different tissue types were included in the study:

- nontumor specimen: skin, liver, colon, kidney, greater omentum;
- tumor specimen: mesothelioma and liver metastasis from colon cancer.

Samples were chosen to represent a wide variety of common tissue samples and settings (nontumor vs tumor) that occur frequently in pathology practice. Samples were up to 1 cm in length and width and 1–3 mm in height.

After harvesting, samples were cut to size and immediately frozen and stored at −20 °C. Thawing of the tissue was performed by wrapping it in saline-soaked gauze.

### 2.3. Instrument

This study was conducted on the RS-G4 Upright (Caliber I.D. Rochester, NY, USA, distributed in Europe by MAVIG GmbH, Munich, Germany).

The RS-G4 is an upright research confocal microscope built to scan large-scale mosaics up to 120 × 80 mm with a maximum resolution of 1024 × 1024 pixels at 5.9 frames per second. The operating wavelengths are 405 nm, 488 nm, 561 nm, 640 nm (all for fluorescence) and 785 nm (reflectance). All available wavelengths were employed except for 405 nm.

### 2.4. Reagents and Staining Protocol

Seven dyes were tested for their ability to depict the ECM in a superior fashion, compared to the instrument’s standard set-up, which employs the fluorescent dye acridine orange for staining nuclei and the so-called reflectance, which is based on the amount of light that is reflected by a surface, for visualizing unstained tissue components like the ECM or the cytoplasm of cells.

All potentially suitable dyes (COL-F, Direct Red 80, Fast Green FCF, Methyl blue, WGA, PAS and eosin) were chosen based on promising results mentioned in the literature or their proven ability to stain the ECM in conventional histology, as well as their fluorescence features with excitation and emission wavelengths compatible with the microscope lasers [8,9,10,11,12,13]. Specifically, we chose PAS and WGA for their expected ability to highlight basement membranes, as well as eosin, which is the standard stain in pathology that pathologists are used to. The other stains were chosen based on literature reports, as described above.

**Figure 1:**
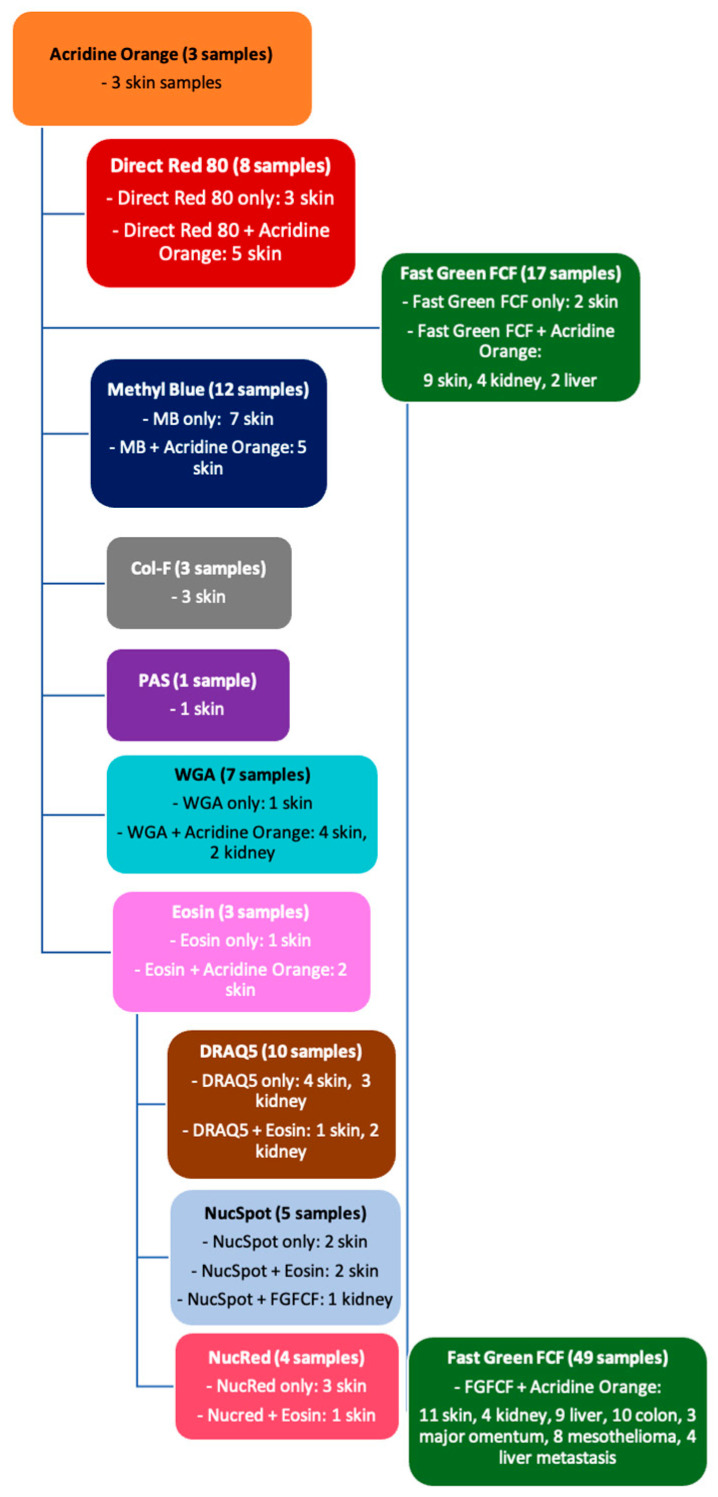
Flowchart of staining process: order of stains and amount and types of tissue samples which were stained.

All samples were incubated in the respective dye (with or without previous immersion in 96% ethanol) and then rinsed in PBS for 15 s (Figure 1).

### 2.5. Staining and Processing

For staining tissue samples, plastic molds (Tissue-Tek^®^ Cryomold^®^ from Sakura Finetek, Staufen im Breisgau, Germany) were used that were 15 × 15 × 5 mm (1.125 mL) and 25 × 20 × 5 mm (2.5 mL). The plastic molds were filled with the required stain or reagent and the tissue sample was immersed into the mold by forceps. During the staining, the tissue was lightly stirred in the mold with the forceps.

### 2.6. Staining Protocols

For testing purposes, staining time and reagent dilutions varied. The rinsing time in PBS was always 15 s.

### 2.7. ECM Stains

#### 2.7.1. Direct Red 80

Incubation for 10 min in Direct Red 80 (1:30 dilution in PBS), then rinsing in PBS, immersion in HCl for 2 × 1 min and rinsing again in PBS.

#### 2.7.2. Fast Green FCF

Incubation in Fast Green FCF (1:30 dilution in PBS) for 90 s, followed by a rinse in PBS.

#### 2.7.3. Methyl Blue

Incubation in Methyl blue (1:20 dilution in PBS) for 5 min, followed by a rinse in PBS.

#### 2.7.4. Col-F Collagen Binding Reagent

Incubation in Col-F (200 μL in 1:20 dilution) for 5 min, followed by a rinse in PBS.

#### 2.7.5. PAS

Incubation in periodic acid for 5 min, then rinsing with aqua destillata, followed by immersion in Schiff reagent for 15 min and eventually rinsing under flowing tap water for 5 min.

#### 2.7.6. Wheat Germ Agglutinin (WGA)

Incubation for 10 min in WGA (1:100 dilution in PBS), followed by a rinse in PBS.

#### 2.7.7. Eosin

Incubation for 1 min in eosin, followed by a rinse in PBS.

After testing, samples were processed according to the in-house protocol for regular FFPE specimens. For comparison with frozen section histology, HE-stained frozen sections were cut from the samples processed for FCM and additionally from similar but unrelated tissue specimens that were entirely untreated.

#### 2.7.8. Nuclear Stains

Acridine orange:

Immersion in 96% ethanol for 30 s, followed by 1 min incubation in acridine orange (1:4 dilution with PBS) and then rinsing in PBS. For testing purposes, the protocol was also used without ethanol.

#### 2.7.9. DRAQ5™

Incubation for 10 min in DRAQ5 (1:10 dilution in PBS), followed by a rinse in PBS.

#### 2.7.10. NucSpot^®^ Live 488

Incubation for 10 min in NucSpot (1 µL/mL PBS), followed by a rinse in PBS.

#### 2.7.11. NucRed^®^ Live 647

Incubation for 5 min in NucRed (4 drops/mL PBS), followed by a rinse in PBS.

### 2.8. Combination of ECM and Nuclear Stainings

In order to achieve an optimized protocol for dual staining, the protocols outlined above were adjusted regarding staining time, dilution of stains and staining order. 

#### 2.8.1. Acridine Orange + Direct Red 80

Ten min incubation in Direct Red 80, rinsing in PBS, 2 min incubation in 0.1% HCl, 45 s immersion in 96% ethanol followed by 45 s incubation in acridine orange (1:4 dilution with PBS) and eventually rinsing in PBS.

Variations of the protocol were tested as ethanol and/or HCl incubation steps were omitted.

#### 2.8.2. Acridine Orange + Fast Green FCF

Incubation for 90 s in Fast Green FCF (1:30 dilution in PBS), rinsing in PBS, and then incubation in acridine orange (1:4 dilution with PBS) for 1 min and eventually rinsing in PBS. Immersing the sample in 96% ethanol before staining with acridine orange was tested. Blending acridine orange and Fast Green FCF in one mold and eventually incubating for 3 min was also tested.

#### 2.8.3. Acridine Orange + Methyl Blue

Incubating for 7 min in Methyl blue (1:3 dilution in PBS), rinsing in PBS, incubating for 45 s in acridine orange (1:4 dilution in PBS) and eventually rinsing in PBS. 

#### 2.8.4. Acridine Orange + PAS

The above-mentioned PAS protocol was used, followed by immersion in 96% ethanol and incubation for 45 s in acridine orange (1:4 dilution in PBS) and eventually rinsing in PBS.

#### 2.8.5. Acridine Orange + WGA

The above-mentioned protocol was used, followed by incubation in acridine orange (1:4 dilution in PBS) for 45 s and eventually rinsing in PBS.

#### 2.8.6. Acridine Orange + Eosin

Incubation for 45 s in eosin followed by rinse in PBS, and then immersion for 45 s in 96% ethanol, followed by 45 s incubation in acridine orange (1:4 dilution in PBS) and eventually rinsing in PBS.

#### 2.8.7. DRAQ5 + Eosin

Incubation for 5 min in DRAQ5 (1:4 dilution in PBS), rinsing in PBS, followed by incubation in eosin for 30 s, and then followed by a rinse in PBS.

#### 2.8.8. NucSpot^®^ Live 488 + Eosin

Incubation for 5 min in NucSpot (1 µL/mL PBS), rinse in PBS, then incubation for 30 s in eosin and eventually rinsing in PBS.

#### 2.8.9. NucSpot^®^ Live 488 + Fast Green FCF

Incubation for 3 min in NucSpot (1 µL/mL PBS), rinse in PBS, incubation for 3 min in Fast Green FCF (1:30 dilution in PBS) and eventually rinsing in PBS.

#### 2.8.10. NucRed^®^ Live 647 + Eosin

Incubation for 5 min in NucRed (8 drops/mL PBS), a rinse in PBS, then incubation for 30 s in eosin and eventually rinsing in PBS.

After testing, samples were processed according to the in-house protocol for regular FFPE specimens. For comparison with frozen section histology, H&E-stained frozen sections were cut from the samples processed for FCM and additionally from similar but unrelated tissue specimens that were entirely untreated.

### 2.9. Image Evaluation

For evaluation and comparison, all physical slides were scanned (PANORAMIC 250 Flash III, 3D Histech) and all images acquired with the RSG4 were transferred to SlideViewer 2.5 (3DHISTECH Ltd., H-1141 Budapest, Oev u. 3., Hungary).

The images were separately analyzed by two pathologists. The analysis was performed based on the following parameters:

(1) Nuclear morphology;
(2) Cell borders;
(3) Texture of the extracellular matrix;
(4) Demarcation of the parenchyma and stroma;
(5) Structural integrity;
(6) Perceptibility of key structures (blood vessels, connective tissue septa or borders of compartments);
(7) Borders of the tissue specimen.

Each parameter was separately scored by two pathologists on a scale from 1 (inadequate) to 5 (comparable to traditional FFPE H&E sections).

Whole-slide images from frozen sections were included as well to compare our findings to the current standard of rapid histologic tissue analysis. For frozen section comparison, we analyzed 3 skin samples, 3 kidney samples, 2 liver samples, 3 adipose tissue samples, 1 colon sample and 1 adenocarcinoma of the colon sample. (A detailed graphical representation of the parameter analyses can be found in Supplementary Figures S1–S7).

### 2.10. Statistics

Sample characteristics were reported on by applying the appropriate method according to the type of value: mean (standard deviation, SD), median (Interquartile ranges, IQRs) or absolute numbers. All statistical analyses were performed using the most recent version at the time of analysis of SPSS (IBM SPSS^®^ Statistics 27) and R (R 4.2.3 for Mac).

## 3. Results

### 3.1. Evaluation of ECM Stains

COL-F, Direct Red 80, eosin, Fast Green FCF, Methyl blue, PAS and WGA were all considered potential candidates for ECM staining based on the criteria mentioned above. During initial testing, eosin showed the most promising results with regard to staining all parts of the ECM in an even manner, with the additional benefit of looking familiar to the pathologist’s eye. It unfortunately proved to be entirely incompatible with the nuclear stain acridine orange, which was by far superior to all other nuclear stains tested and therefore was the only choice for staining nuclei. Other stains showed inhomogeneous staining patterns, e.g., PAS and WGA, and were excluded from further testing (for an overview of all evaluations see Table 1).

The above-mentioned evaluation and selection of stains resulted in the following protocol: staining the tissue sample for 90 s in Fast Green FCF (1:30 dilution in PBS), rinsing it in PBS, then staining it with acridine orange (1:4 dilution with PBS) for 1 min and eventually rinsing it in PBS.

### 3.2. Overall Representation of ECM Texture in Connective Tissue with Fast Green FCF

**Figure 2:**
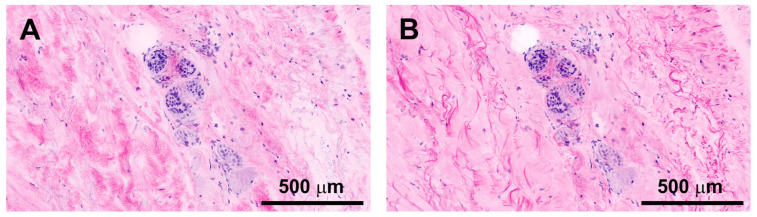
Comparison of FCM: acridine orange + reflectance (**A**) and CM: acridine orange + Fast Green FCF (**B**) in sweat glands within the dermis of the skin.

The fibrillar structure of the ECM, which appeared cloudy and speckled in acridine orange + reflectance, was well defined with the addition of Fast Green FCF (Figure 2). A higher magnification confirmed this impression. The ECM structures presented were better defined, improving the visibility and identifiability of the borders between cells and the ECM in comparison to staining with acridine orange + reflectance. The overall ECM staining in acridine orange + Fast Green FCF seemed more homogenous and less blotty than it did in acridine orange + reflectance.

Another noticeable difference was the more intense visualization of elastic fibers in addition to collagen bundles in acridine orange + Fast Green FCF as both stains, especially Fast Green FCF, stained them (Figure 2).

The depiction of nuclei was unaffected by Fast Green FCF.

**Figure 3:**
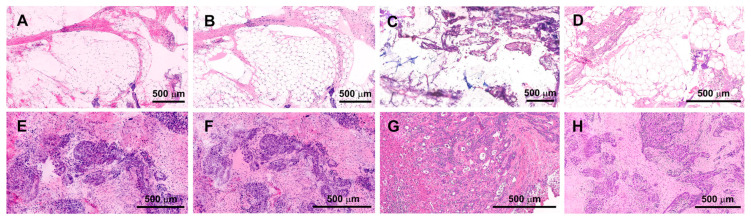
Comparison of FCM: acridine orange + reflectance (**A**,**E**); CM: acridine orange + Fast Green FCF (**B**,**F**), frozen section, (**C**,**G**) FFPE and (**D**,**H**) HE. (**A**–**D**) major omentum and (**E**–**H**) mesothelioma.

Following this selection, FCM employing Fast Green FCF was systematically compared to FCM without the addition of Fast Green FCF based on the seven tissue parameters described above in an attempt to quantify the differences (Figure 3).

### 3.3. Display of Cellular Details and Other Tissue Structures

In an attempt to quantify the differences between the two staining procedures, we scored seven key morphologic parameters (nuclear morphology, cell borders, texture of ECM, demarcation of parenchyma and stroma, structural integrity, perceptibility of key structures and borders of the tissue specimen) as described above.

Notably, AO + FGFCF received higher scores than AO + reflectance in most/all tissue samples in the categories “texture of ECM”, “demarcation of parenchyma and stroma” as well as “perceptibility of key structures”. The structural integrity and visibility of the borders of the tissue specimen were equivalent in both procedures, while the demarcation of cell borders showed better scoring for FGFCF only in adipose tissue and the colon sample. 

When comparing FCM to frozen sectioning, both AO + FGFCF and AO + reflectance received consistently higher scores for all parameters in adipose tissue.

For the other types of tissues, we found that FCM performed better in the categories of structural integrity (except for kidney), perceptibility of key features (except liver) and tissue borders (except kidney). Frozen sectioning, on the other hand, performed better for the parameters demarcation of parenchyma (except liver) and depiction of cell borders.

Both FCM and frozen sectioning displayed mostly equivalent performance for nuclear morphology; frozen sectioning did, however, perform worse in liver, liver metastasis and colon samples. 

The morphologic detail of the extracellular matrix was best represented by AO + FGFCF in adipose tissue, skin, liver and mesothelioma, while it was roughly equivalent in the other tissues.

### 3.4. Total Processing Time for CM Was Generally Short

The processing time for tissue samples stained with acridine orange + Fast Green FCF was approximately 2 min for staining and approximately 1 min for accurate slide covering and positioning onto the stage holder of the microscope. Scanning and post-processing time depended on the size of the tissue sample, between 5 and 20 min (in rare cases).

## 4. Discussion

The major strength of ex vivo FCM is the ability to perform nondestructive microscopic assessments of tissues without prior fixation or other kinds of conventional histologic workup. The whole procedure can be completed within minutes, a time frame comparable to conventional frozen section diagnostics. In addition, FCM avoids the tissue damage associated with freezing and is performed without loss of tissue by sectioning. It also does not require a specially equipped histology laboratory with adequately trained staff. 

There are several areas where ex vivo FCM might be a valuable addition or even alternative to (frozen section) histology: the non-destructive microscopic investigation of small tissue samples in both research and diagnostics, which includes potential assessment of tissue adequacy prior to further workup with conventional methods. It might also serve as an option for intraoperative diagnostic histology at institutions that do not have frozen section histology services available on-site.

For these reasons, ex vivo CM is currently being studied intensely, and is already applied in some fields, such as dermatology and urology, as a feasible and fast method for tissue evaluation without the need for fixation [2,3,4,5,14,15,16,17,18].

By using acridine orange as a nuclear stain and a reflectance laser for depiction of other tissue components, a realistic presentation of different tissue types can be created digitally within a short time frame. The images are digitally colored in order to achieve an appearance similar to H&E-stained histology slides, thereby rendering them ideal for pathologists.

However, despite the familiar-looking staining pattern, we found in a previous study that the visualization of the ECM by reflectance laser was unsatisfactory when examined in greater detail [6]. Although the morphology of nuclei was excellent in FCM, the relationship of cells to their surroundings was not always well defined. In particular, it was often difficult to identify the type and structure of connective tissue as staining intensity in reflectance does not necessarily reflect tissue density, as would be the case in conventionally stained tissue sections. The pattern and distribution of staining signals in reflectance also differ from histologic sections as they often do not match the texture of connective tissue in H&E staining. Especially when considering implementation of this novel technique to cancer diagnostics, e.g., the evaluation of the invasiveness of a tumor, a reliable representation of the ECM is crucial.

Our selection process revealed that a combination of acridine orange and Fast Green FCF yielded the best overall results, with improved ECM depiction in several organ samples and a depiction of structures comparable to HE -stained histology sections. 

Given the fact that acridine orange + Fast Green FCF outperformed acridine orange + reflectance in almost every parameter (most markedly in “presentation of the extracellular matrix” and particularly in adipose tissue), this staining combination holds potential both for research and clinical application:

the better depiction of the extracellular matrix might be of value in every application where the structure and amount of fibrotic tissue is of interest, e.g., fibrosis/desmoplasia in the context of a tumor or in chronic inflammation.

Other features of this combination were a short staining time (ca. 2 min) and a simple staining protocol, which would make it a feasible method for rapid tissue analysis. We therefore compared FCM to frozen sections. In terms of morphologic detail, FCM was indeed equivalent or even clearly superior (in adipose tissue) except for the visibility of cell borders and the demarcation of parenchyma and stroma.

A notable point in the design of this study was the use of the RS-G4 microscope, which, while ideal for identifying novel staining combinations, is mainly designed for research purposes and not for clinical diagnostics. With its additional functions and modalities (such as a variety of lasers and laser settings), the duration of image processing was longer than it would usually be on a device intended for clinical use.

Another limitation of this study is the amount and somewhat limited selection of tissue samples, as the study was conducted on left-over tissue from routine pathology that was not needed for further analysis.

Of note is also the fact that an important target structure that could not be specifically highlighted by our method was the basement membranes. This fact might be of relevance for the detection of early phases of tumor invasion.

## 5. Conclusions

This study shows that by using the ECM stain Fast Green FCF in combination with the nuclear stain acridine orange, the histologic features of different tissue types can be displayed satisfactorily and, depending on tissue type, in a superior fashion to acridine orange + reflectance by CM.

Our proposed method improves not only the depiction of the ECM in comparison to acridine orange + reflectance but also the overall representation of tissue structures; especially in adipose tissue, the improvements are of note. The protocol we developed is fast and easy to perform.

The total processing time is short enough for a potential diagnostic application, especially in the setting of intraoperative microscopy.

## Figures and Tables

**Table 1:** Summary of the evaluation of all stains tested.

ECM Stains	Staining Result	Final Selection	Reason for Selection
Direct Red 80	Decent ECM staining	×	Overlaying nuclear stain, inhomogeneities
Fast Green FCF	Satisfying ECM staining	✓	Detailed ECM staining, short staining time, compatible with acridine orange
Methyl Blue	ECM staining, but less defined than *Fast Green FCF*	×	Varying staining intensity, smearing
COL-F	Decent ECM staining	×	Only excitable on the same wavelength as acridine orange
PAS	ECM staining, partially basal lamina staining	×	Irregular staining pattern
WGA	ECM staining, partial basal lamina staining	×	Inhomogenous staining pattern
Eosin	Satisfying ECM staining	×	Not compatible with acridine orange
**Nuclear Stains**			
Acridine Orange	Satisfactory nuclear depiction	✓	Decent nuclear detail, short staining time
DRAQ5	Nuclei were stained, but only using high laser power and PMT	×	Blurred nuclei in pseudocolor mode
Nucspot	Decent nuclear depiction	×	Not compatible in combination with eosin
Nucred	Nuclei were stained, but only using high laser power and PMT	×	Blurred nuclei in pseudocolor mode

## Data Availability

Data will be made available by the corresponding author upon reasonable request.

## Supplementary Materials

The following supporting information can be downloaded at: https://www.mdpi.com/article/10.3390/life14101240/s1, Figure S1: Scores of parameter “nuclear morphology”; Figure S2: Scores of parameter “cell borders”; Figure S3: Scores of parameter “presentation of extracellular matrix”; Figure S4: Scores of parameter “demarcation of parenchyma and stroma”; Figure S5: Scores of parameter “structural integrity”; Figure S6: Scores of parameter “perceptibility of key structures”; Figure S7: Scores of parameter “tissue borders”.
