# Supplementary material for: Fast Green FCF Improves Depiction of Extracellular Matrix in Ex Vivo Fluorescence Confocal Microscopy
Source: Life (Basel). 2024 Sep 28;14(10):1240. doi: 10.3390/life14101240 (PMC11508659; doi:10.3390/life14101240)
Supplement: Supplementary file 1 [file life-14-01240-s001.zip › life-3194066-supplementary.pdf]

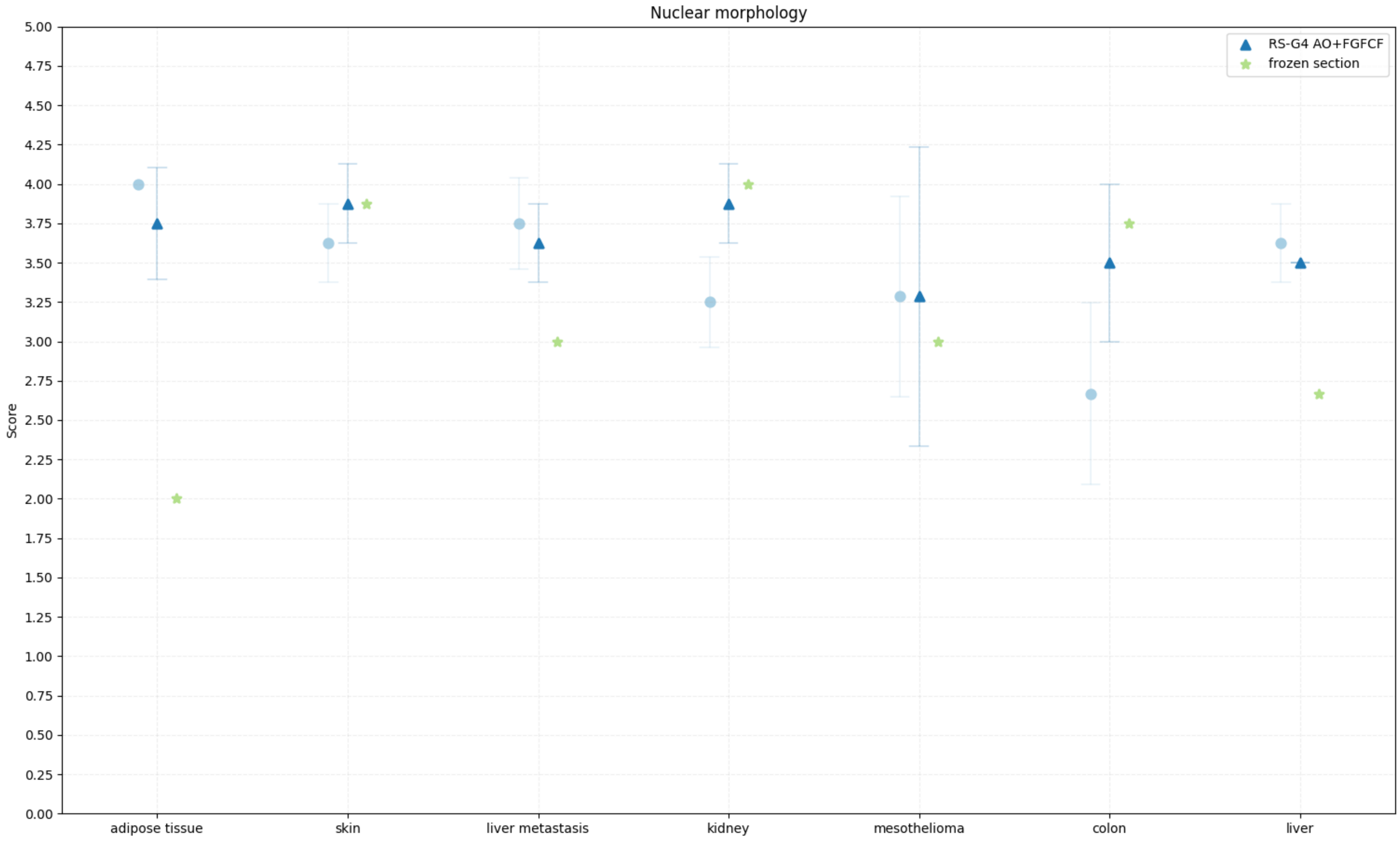

Nuclear morphology
RS-G4 AO+FGFCF
frozen section
Score
5.00
4.75
4.50
4.25
4.00
3.75
3.50
3.25
3.00
2.75
2.50
2.25
2.00
1.75
1.50
1.25
1.00
0.75
0.50
0.25
0.00
adipose tissue
skin
liver metastasis
kidney
mesothelioma
colon
liver

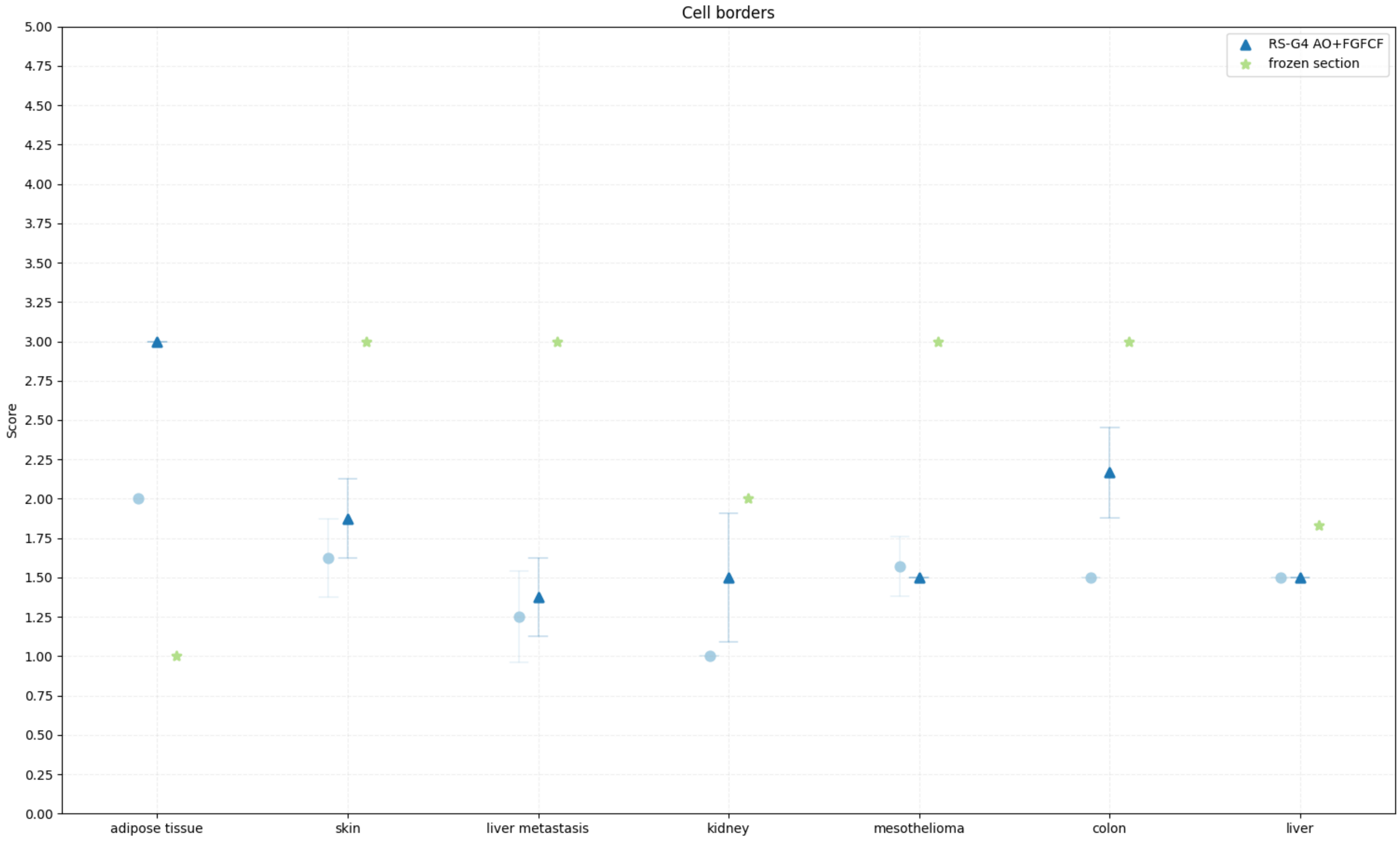

Cell borders
Score
RS-G4 AO+FGFCF
frozen section
5.00
4.75
4.50
4.25
4.00
3.75
3.50
3.25
3.00
2.75
2.50
2.25
2.00
1.75
1.50
1.25
1.00
0.75
0.50
0.25
0.00
adipose tissue
skin
liver metastasis
kidney
mesothelioma
colon
liver

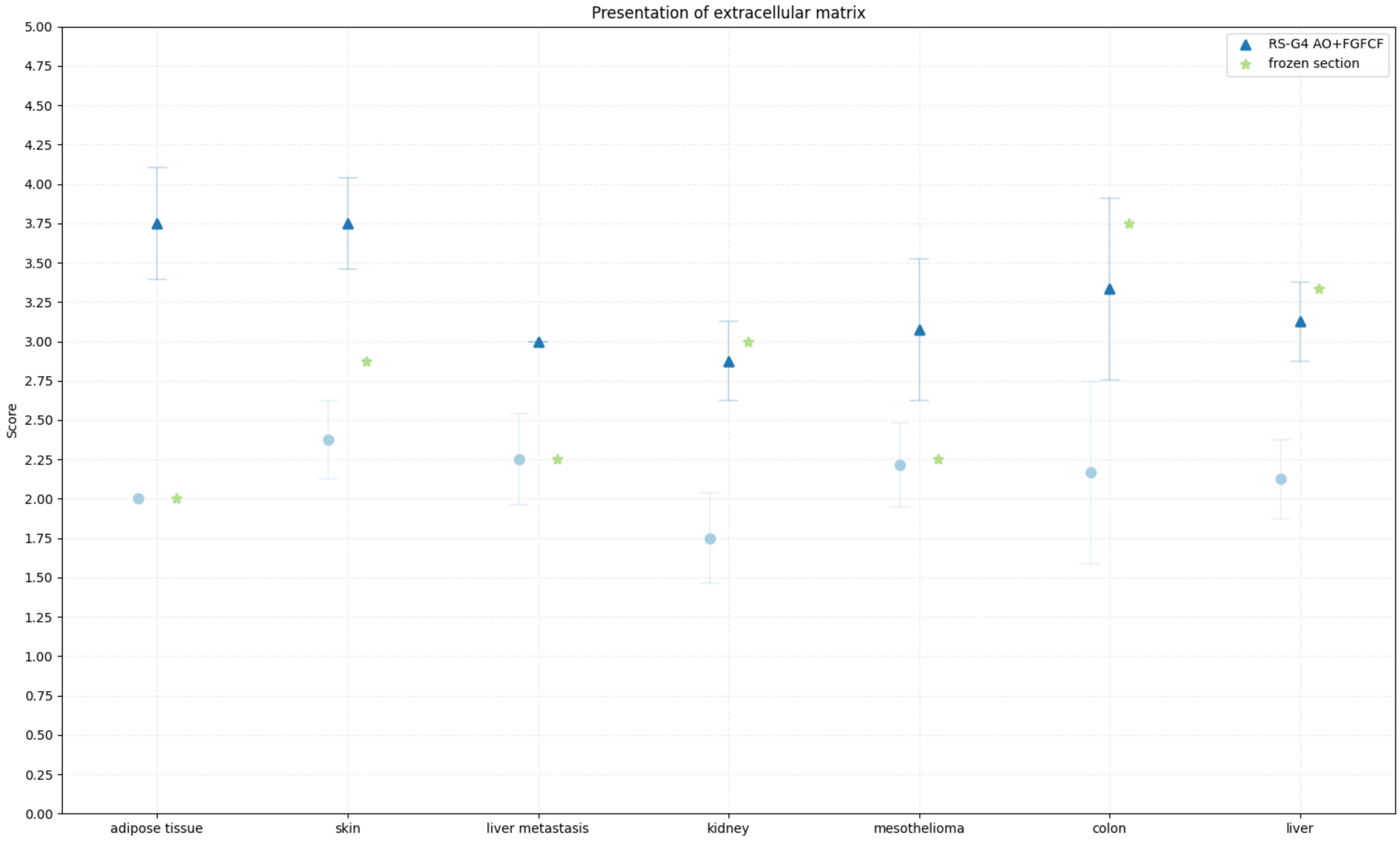
Presentation of extracellular matrix
RS-G4 AO+FGFCF
frozen section
Score
5.00
4.75
4.50
4.25
4.00
3.75
3.50
3.25
3.00
2.75
2.50
2.25
2.00
1.75
1.50
1.25
1.00
0.75
0.50
0.25
0.00
adipose tissue
skin
liver metastasis
kidney
mesothelioma
colon
liver

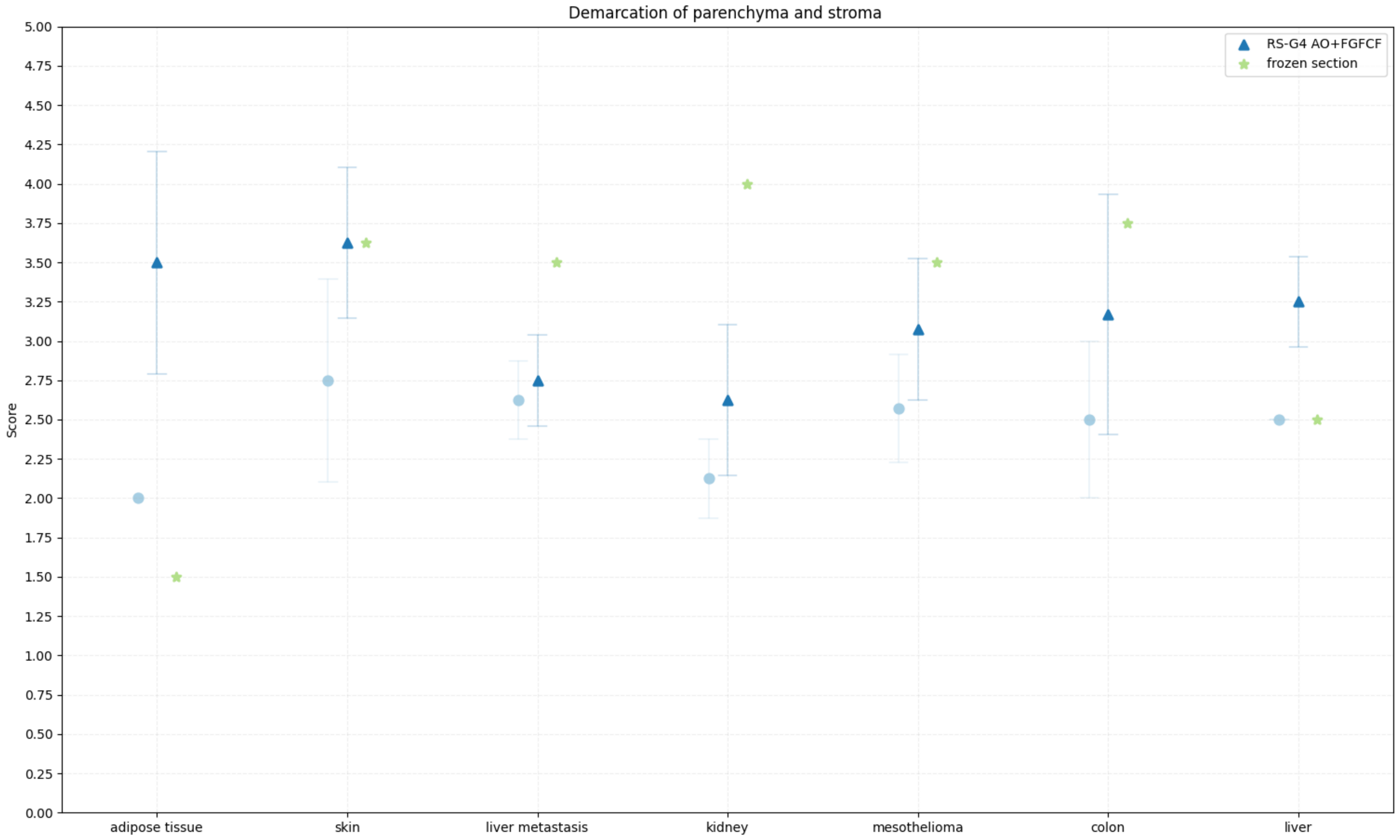

Demarcation of parenchyma and stroma
RS-G4 AO+FGFCF
frozen section
Score
5.00
4.75
4.50
4.25
4.00
3.75
3.50
3.25
3.00
2.75
2.50
2.25
2.00
1.75
1.50
1.25
1.00
0.75
0.50
0.25
0.00
adipose tissue
skin
liver metastasis
kidney
mesothelioma
colon
liver

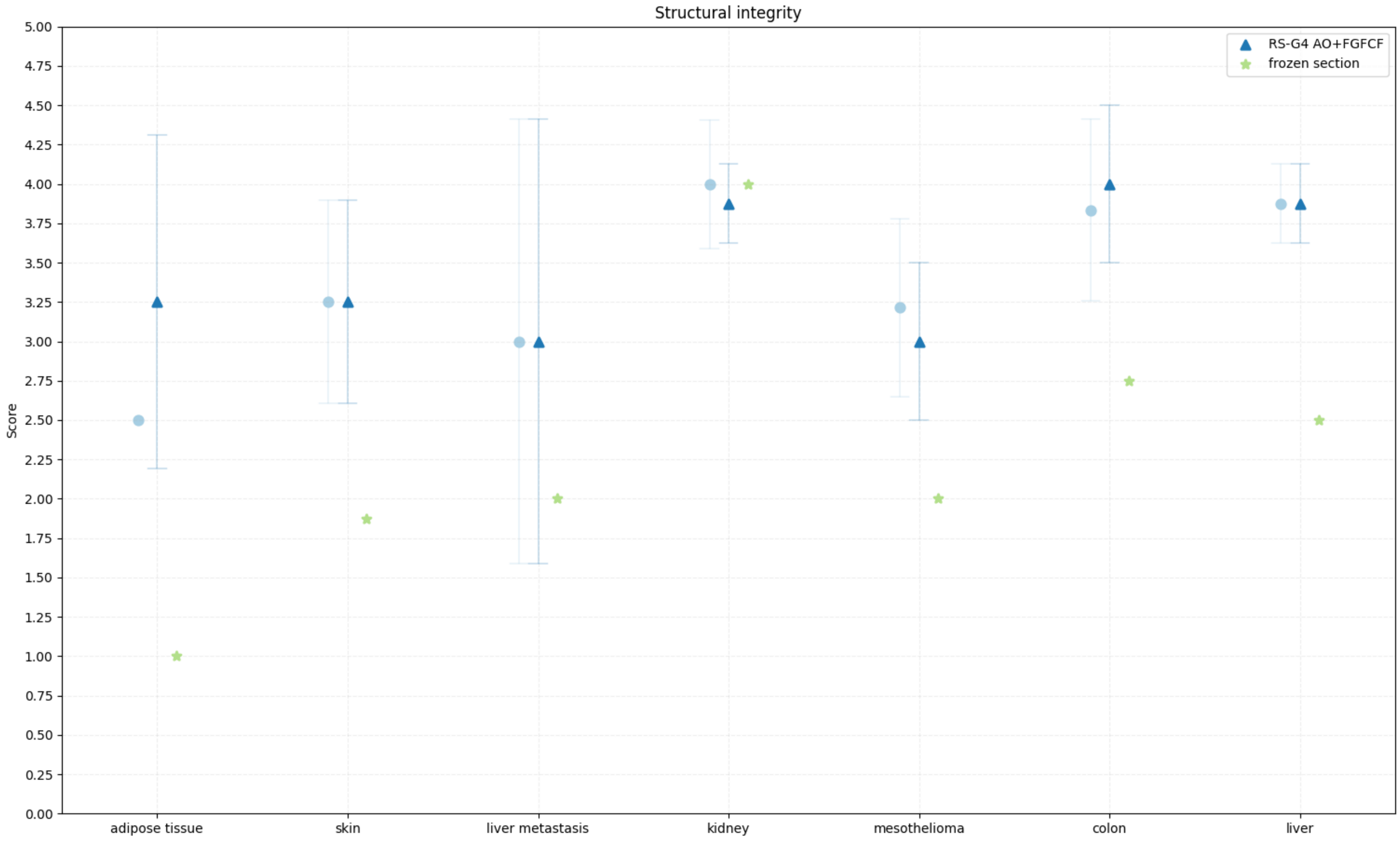

Structural integrity
Score
RS-G4 AO+FGFCF
frozen section
5.00
4.75
4.50
4.25
4.00
3.75
3.50
3.25
3.00
2.75
2.50
2.25
2.00
1.75
1.50
1.25
1.00
0.75
0.50
0.25
0.00
adipose tissue
skin
liver metastasis
kidney
mesothelioma
colon
liver

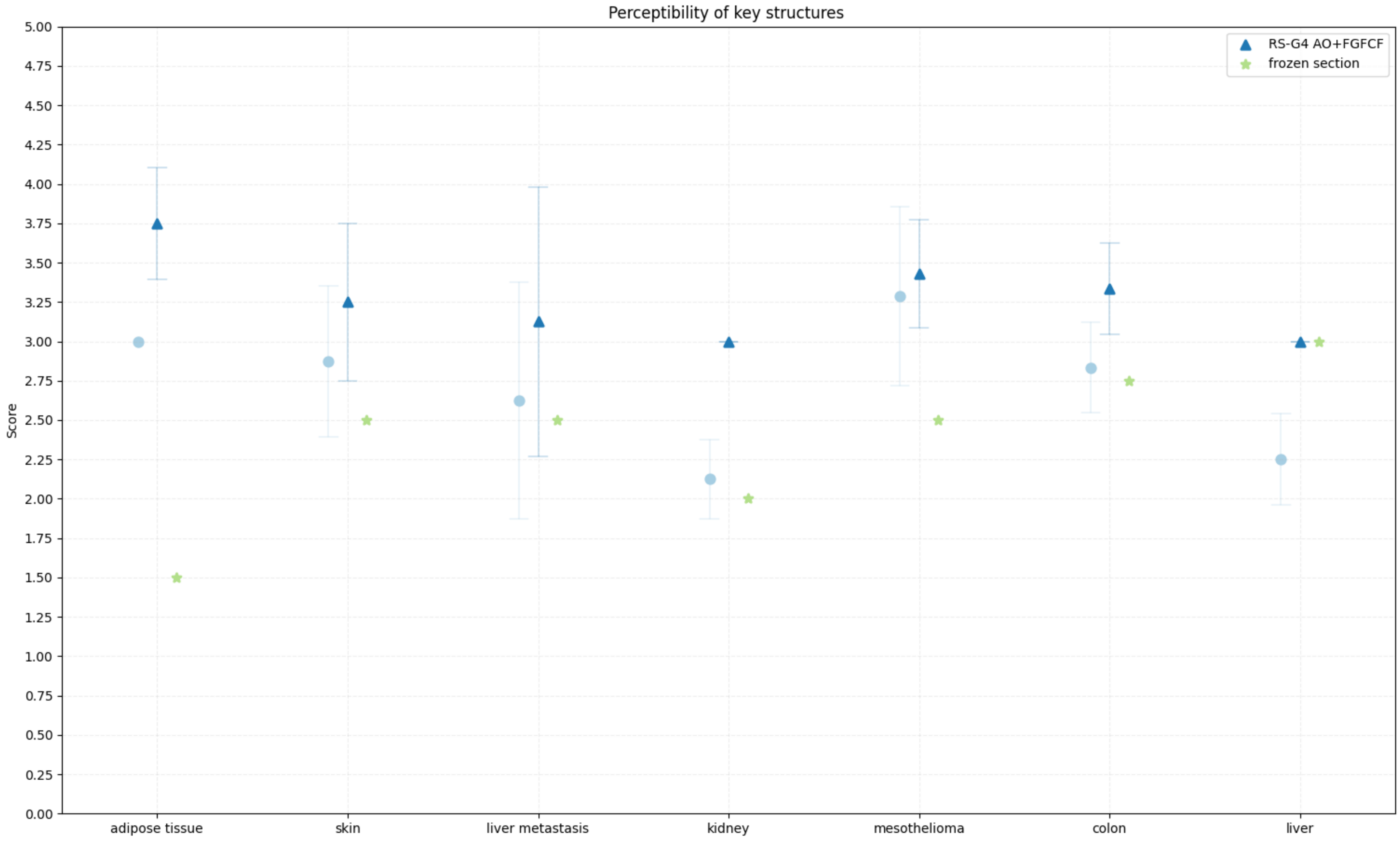

Perceptibility of key structures
RS-G4 AO+FGFCF
frozen section
Score
5.00
4.75
4.50
4.25
4.00
3.75
3.50
3.25
3.00
2.75
2.50
2.25
2.00
1.75
1.50
1.25
1.00
0.75
0.50
0.25
0.00
adipose tissue
skin
liver metastasis
kidney
mesothelioma
colon
liver

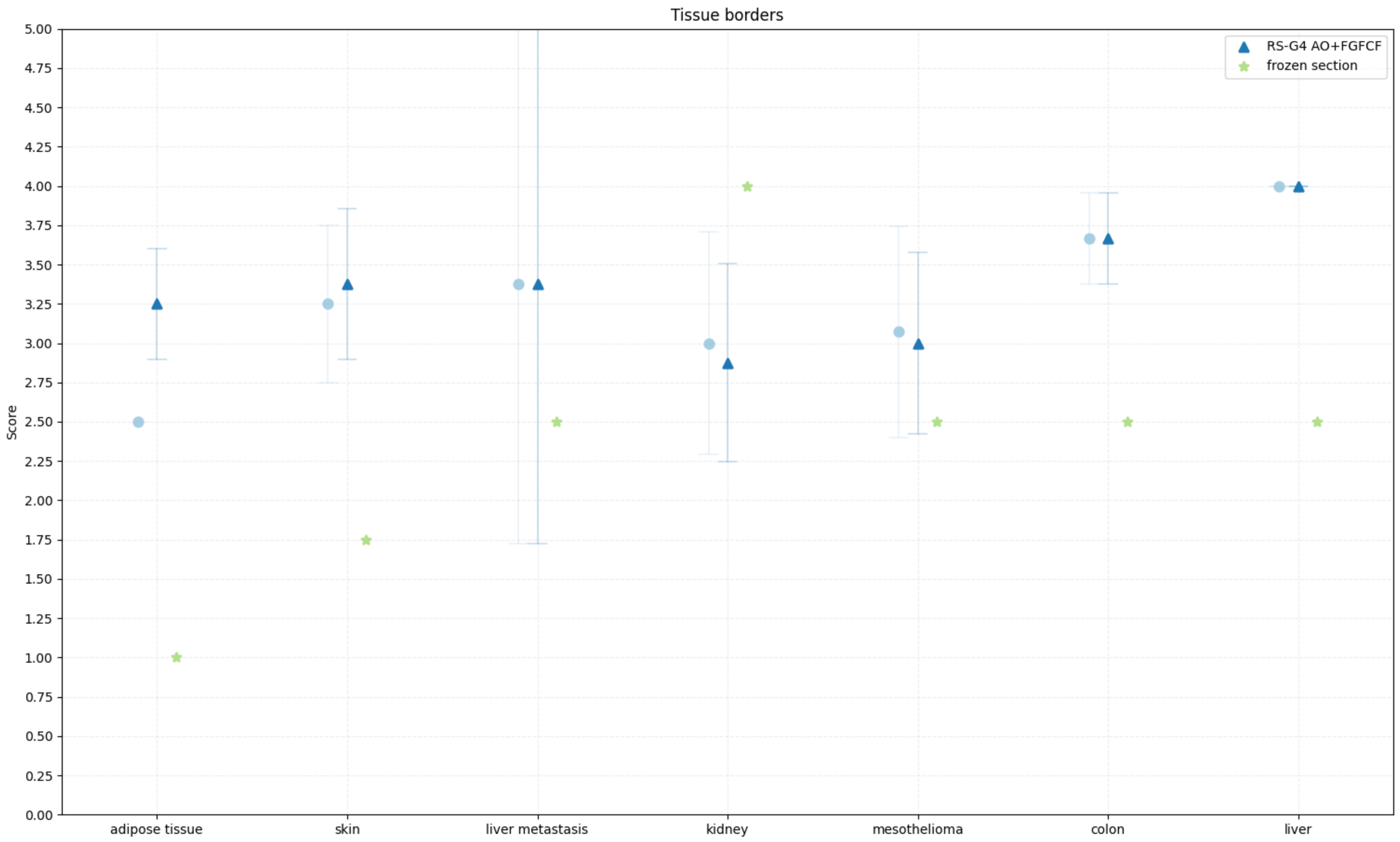
Tissue borders
RS-G4 AO+FGFCF
frozen section
Score
5.00
4.75
4.50
4.25
4.00
3.75
3.50
3.25
3.00
2.75
2.50
2.25
2.00
1.75
1.50
1.25
1.00
0.75
0.50
0.25
0.00
adipose tissue
skin
liver metastasis
kidney
mesothelioma
colon
liver

Figure caption supplementary figures

Supplementary Figure S1 Scores of parameter “nuclear morphology”

Supplementary Figure S2 Scores of parameter “cell borders”

Supplementary Figure S3 Scores of parameter “presentation of extracellular matrix”

Supplementary Figure S4 Scores of parameter “demarcation of parenchyma and stroma”

Supplementary Figure S5 Scores of parameter “structural integrity”

Supplementary Figure S6 Scores of parameter “perceptibility of key structures”

Supplementary Figure S7 Scores of parameter “tissue borders”
